# Quality of life in polyneuropathy: association with biomarkers of small fiber impairment

**DOI:** 10.1186/s12955-015-0363-9

**Published:** 2015-10-15

**Authors:** Meng-Ting Lin, Lukas Jyuhn-Hsiarn Lee, Chi-Chao Chao, Sung-Tsang Hsieh

**Affiliations:** School of Medicine, National Taiwan University College of Medicine, Taipei, Taiwan; Department of Neurology, National Taiwan University Hospital, 7 Chung-Shan S Road, Taipei, 10002 Taiwan; National Institute of Environmental Medicine Sciences, National Health Research Institutes, 35 Keyan Road, Zhunan, 35053 Taiwan; Department of Anatomy and Cell Biology, National Taiwan University College of Medicine, Taipei, 10051 Taiwan; Graduate Institute of Brain and Mind Sciences, National Taiwan University College of Medicine, Taipei, 10051 Taiwan

**Keywords:** Quality of life, Quantitative sensory testing, Skin biopsy, Small fiber neuropathy, Polyneuropathy

## Abstract

**Background:**

Polyneuropathy presumably lowers quality of life (QoL). However, there is a lack of systematic studies that assess QoL changes and biomarkers of polyneuropathy as determinants of QoL. We aimed to investigate the relationship between every specific aspect of QoL and the clinical parameters used to assess the impairment of motor, sensory (large and small fibers), and autonomic nerves in polyneuropathy.

**Methods:**

Polyneuropathy patients were recruited from September 2013 to March 2014; QoL was assessed using (1) the WHO Quality of Life-BREF (WHOQoL), (2) the European Quality of Life-5 Dimensions, and (3) the Brief Pain Inventory Short Form. Neuropathy examinations included nerve conduction studies, autonomic function tests, quantitative sensory testing (QST), and intraepidermal nerve fiber (IENF) density assessment of skin biopsies.

**Results:**

There were 61 polyneuropathy patients (male/female = 38/23, mean age 58.14 ± 12.95 years). Patients had a lower QoL than age-and gender-matched controls in the physical and psychological domains of the WHOQoL. Among the biomarkers for different nerve fiber categories, only the small fiber neuropathy assessments were significantly related to all domains of the WHOQoL. In contrast, the parameters of the large fiber neuropathy were independent of QoL. Patients with abnormal temperature thresholds and a lower IENF density had lower WHOQoL scores compared to patients with normal thresholds and IENF densities. Warm threshold of the foot in QST was linearly correlated with all domains of the WHOQoL.

**Conclusions:**

QoL scores were reduced in polyneuropathy, and biomarkers of small fiber neuropathy, i.e., warm threshold and IENF density were discriminating predictors of QoL.

**Electronic supplementary material:**

The online version of this article (doi:10.1186/s12955-015-0363-9) contains supplementary material, which is available to authorized users.

## Background

Polyneuropathy resulting from different etiologies, such as diabetes, autoimmune diseases, and hereditary neuropathy, causes various impairments and presumably influences quality of life (QoL) [1, 2]. The measurements of QoL provide quantitative and functional assessments of these relations [3]. The extensive involvement of nerve fibers with different types of polyneuropathy usually leads to impaired physical activities and symptoms interfering daily living, such as reduced muscle strength and neuropathic pain [4].

Studies investigating the QoL in polyneuropathy cohorts, however, have been limited and are often inconclusive [3, 4]. According to pathology patterns, physiological features, and clinical symptoms, polyneuropathy can be categorized into large and small fiber types. Symptoms of large fiber neuropathy include weakness of limbs and a loss of proprioception resulting in sensory ataxia, which are evaluated using conventional nerve conduction studies (NCS). Symptoms of small fiber neuropathy include reductions in temperature sensations and autonomic functions with frequent neuralgia [5]. Traditionally, the assessment and diagnosis of small fiber neuropathy has primarily depended on the subjective description of symptoms [6]. To facilitate accurate diagnosis, several groups, including ours, have established quantifiable parameters for small fiber neuropathy, i.e., temperature thresholds determined by quantitative sensory testing (QST) and intraepidermal nerve fiber (IENF) density of skin biopsies [7, 8].

Peripheral neuropathy has been associated with increased mortality in diabetic patients [9]. Neurological impairments were previously assessed using measures of health-related QoL (HRQoL) [10], which provides evaluations on health status, i.e., disease impacts on health-related domains of QoL. A previous comprehensive review linked HRQoL to clinical variables, formulating a conceptual model which included five domains of patient reported outcomes: biological and physiological factors, symptoms, functioning, general health perceptions, and overall QoL [11]. Previous studies have mainly used the HRQoL measure SF-36 or disease-specific measures of HRQoL, such as PN-QoL-97 or NeuroQoL [12], and only a few studies have investigated global links to QoL [13]. Thus, the influences of polyneuropathy on the global QoL have not yet been systematically examined. In this study, we used the WHO Quality of Life-BREF (WHOQoL), a measure of global QoL to evaluate the associations of polyneuropathy with QoL. The aim of our study was to investigate the relationship between every specific aspect of global QoL and biomarkers of polyneuropathy, including large and small fiber nerves, i.e., parameters of nerve conduction studies, autonomic function tests, QST, and skin biopsy.

## Methods

### Patient enrollment

Patients with polyneuropathy were recruited from the Department of Neurology, National Taiwan University Hospital, Taipei, Taiwan from January 2013 to March 2014.

The recruitment was consecutive case series where board-certificated neurologist examined all the patients with standard approach. Polyneuropathy was diagnosed according to (1) a symmetric and length-dependent symptoms and signs in the motor or sensory systems [14], and (2) abnormality in nerve conduction study or skin biopsy of the leg suggestive of neuropathy. The neurological examinations followed routine procedures and included detailed assessments of muscle strength and the sensations to heat, cold, pinprick, and vibratory stimuli. Laboratory examinations included the NCS (Additional file 1), QST, autonomic function tests (Additional file 1), and skin biopsy. The underlying etiologies of polyneuropathy were evaluated using a battery of tests for diabetes, endocrine, autoimmune, and genetic neuropathies as previously described [14]. QoL was surveyed using the following questionnaires: (1) the WHO Quality of Life-BREF (WHOQoL), (2) the European Quality of Life-5 Dimensions (EQ-5D), and (3) the Brief Pain Inventory Short Form (BPI-SF).

### Standard protocol approvals, registrations, and patient consents

This study was conducted following the World Medical Association’s Declaration of Helsinki. The protocols were approved by the Institutional Review Board of National Taiwan University Hospital, Taipei, Taiwan (IRB No. 201306078RINC). Patients signed an informed consent document before participation. Procedures of the tests (nerve conduction studies, quantitative sensory testing, autonomic function tests, and skin biopsy for quantitation of epidermal innervaion) followed established protocols and age-and gender-matched control subjects were retrieved from a previously described database [15]. All these were detailed in the following and in the Supplementary Methods.

### World health organization quality of life instrument

We used the WHOQoL questionnaires to assess the global QoL in 4 domains, physical, psychological, social, and environment [13, 16]. To evaluate the global impacts of polyneuropathy, the WHOQoL scores of our patients were compared with scores from gender-and age-matched healthy Taiwanese control subjects (Additional file 1).

### European Quality of Life-5 Dimensions (EQ-5D)

It is a descriptive system to form an index score (EQ index) covering 5 dimensions of HRQoL, mobility, self-care, usual activity, pain/discomfort, anxiety/depression, with a 3-point scale (1 point for ‘no problem’, 2 point for ‘some problem’, and 3 point for ‘extreme problem’) in each dimension [17, 18]. In addition, the EQ-5D also consists of a visual analogue scale (EQ VAS) that ranges from 0 (worst imaginable health state) to 100 (best imaginable health state), which records the respondent’s self-rated evaluation of HRQoL. The scores and index of our patients were compared with those from gender-and age-matched healthy Taiwanese control subjects (Additional file 1).

### Brief pain inventory *short form*

The Brief Pain Inventory short form (BPI-SF) Taiwan version consists of numerical scales (0 ~ 10) for 3 components: (1) pain severity score ‘within 1 week’ (2), pain severity ‘right now’, and (3) pain interference score (the average score from 7 items that represent interference with daily functions, mood, sleep, and enjoyment of life) [19] (Additional file 1).

### Quantitative sensory testing

Quantitative sensory testing (QST) was performed using a Thermal Sensory Analyzer (Medoc Advanced Medical System, Minneapolis, MN) to measure the thermal thresholds for warm and cold sensations using a previously established standard procedure [20]. Thermal thresholds were expressed as a warm threshold (WT) and cold threshold (CT), and were measured at the thenar eminence and index of the hand and at the dorsum and great toe of the foot. Cut-off values were designated as the 95^th^ percentile value (for warm threshold) and the 5th percentile value (for cold threshold) according to our normative database [14]. In this study, the overall QST results were classified as abnormal if at least 1 temperature threshold was beyond the cut-off value (Additional file 1).

### Skin biopsy and quantitation of IENF density

We conducted skin biopsies to provide pathologic evidence of small fiber neuropathy. Following our published procedures, a skin biopsy was performed on the distal leg after an informed consent document was signed [21–23]. Sections of skin biopsies were immunostained and quantified using a previously described method [23] (Additional file 1).

### Study design and statistical analysis

For each examined parameter, patients were classified into two groups, normal and abnormal. The scores for each item of the WHOQoL, EQ-5D, and BPI-SF between these two groups were analyzed accordingly. The parametric t-test or non-parametric Wilcoxon rank-sum test were used depending on whether the data followed a Gaussian distribution or not. Univariate and multivariate linear regression models were used to explore the relationships between each domain of QoL and examination parameter. A P<0.05 was considered statistically significant. SAS 9.3 (SAS Institute Inc, Cary, NC) was used for statistical analyses.

## Results

### Demographic data, clinical presentations, and QoL measures of polyneuropathy

There were 61 polyneuropathy patients (male/female = 38/23) with a mean age of 58.14 ± 12.95 years and a mean disease duration of 4.34 ± 3.26 years. The underlying etiologies consisted of diabetes, immune-related, drug-induced, inherited, and idiopathic types. Neurological examinations assessed the functions of motor system (muscle strength on a MRC grade), sensory and autonomic system. Among small-fiber sensory impairments, proportion of impaired pinprick sensation, burning or tingling sensation and other small fiber characteristics (electric shock–like or cold-like pain) was 80.3 %, 77.0 % and 8.2 % respectively (Table 1).

**Table 1:** Clinical profiles of patients with polyneuropathy

Features	Patients with neuropathy
Men/Women	38/23
Age (Mean ± SD)	58.14±12.95
Mean duration of disease (Interquartile range)	4 (1–6)
Neurological examinations	
Motor system^a^	
Upper limbs	Proximal: 4.82 ± 0.59 / Distal: 4.68 ± 0.58 ^b^
Lower limbs	Proximal: 4.66 ± 0.84 / Distal: 4.42 ± 0.85 ^c^
Sensory system	
Large-fiber sensory impairment	
Impaired proprioception	65.6 %
Impaired vibration sensation at ankles	65.6 %
Small-fiber sensory impairment	
Impaired pinprick sensation	80.3 %
Burning or tingling sensation	77.0 %
Other small fiber characteristics ^d^	8.2 %
Autonomic system	
Gastrointestinal symptoms	32.8 %
Genitourinary symptoms	26.2 %
Orthostatic hypotension	29.5 %
Sudomotor failure	37.7 %
Etiology	
Diabetic polyneuropathy	10 (16.4 %)
Immune-related polyneuropathy ^e^	11 (18.0 %)
Drug-induced polyneuropathy ^f^	4 ( 6.6 %)
* Inherited* polyneuropathy ^g^	10 (16.4 %)
Idiopathic polyneuropathy	26 (42.6 %)

a:Muscle strength: according to Medical Research Council (MRC) grade

b:Proximal muscle of upper limbs included abduction muscle of the shoulder. Distal muscles of upper limbs included flexion and extension muscles of the wrist and grasp of the hand

c:Proximal muscle of lower limbs included hip flexion muscle. Distal muscles of lower limbs included dorsiflexion and plantar flexion muscles of the ankle

d:Electric shock–like or cold-like pain

e:Chronic inflammatory demyelinating polyneuropathy (5), Sjögren syndrome (2), vasculitic neuropathy (2), Guillain-Barré syndrome (1), IgM paraproteinemic neuropathy (1)

f:Chemotherapy induced neuropathy (3): docetaxel (2), cisplatin (1) and metronidazole induced neuropathy (1)

g:Charcot-Marie-Tooth disease (3), familial amyloid polyneuropathy (7)

To evaluate the global relations of polyneuropathy, we surveyed different dimensions of QoL and compared these results with age-and gender-matched healthy individuals. Among the 4 domains of the WHOQoL, polyneuropathy patients had significantly lower scores in the physical domain (48.9 ± 10.8 vs. 68.1 ± 14.8, p < 0.0001) and psychological domain (51.8 ± 13.2 vs. 60.6 ± 14.2, *p*<0.0001). Among EQ-5D, polyneuropathy patients had significantly lower scores in all dimensions, index and VAS (Table 2).

**Table 2:** Comparison of WHOQoL: between polyneuropathy patients and gender- and age-matched healthy subjects

	Polyneuropathy patients		Matched healthy subjects		*P* value (t-test)
	*N*	Score (Mean ± SD)	*N*	Score (Mean ± SD)	
WHOQoL					
Domain 1 (Physical)	61	48.9 ± 10.8	61	68.2 ± 12.5	<.0001
Domain 2 (Psychological)	61	51.8 ± 13.2	61	62.0 ± 13.8	<.0001
Domain 3 (Social)	61	64.7 ± 14.4	61	63.0 ± 12.4	0.4928
Domain 4 (Environment)	61	64.3 ± 14.1	61	62.0 ± 14.3	0.3769
EQ-5D					
Mobility	61	1.56 ± 0.50	61	1.02 ± 0.13	<.0001
Self-care	61	1.26 ± 0.48	61	1.03 ± 0.26	<.0001
Usual activity	61	1.38 ± 0.49	61	1.08 ± 0.28	<.0001
Pain / discomfort	61	1.95 ± 0.46	61	1.25 ± 0.43	<.0001
Anxiety / depression	61	1.30 ± 0.56	61	1.13 ± 0.34	0.089
Index	61	0.51 ± 0.26	61	0.88 ± 0.20	<.0001
VAS	61	63.67 ± 16.23	61	73.56 ± 13.46	<.0001

Abbreviation: *WHOQoL* WHO quality of life-BREF, *EQ-5D* European Quality of Life-5 Dimensions

### Small fiber function: sensory system

To investigate the association between small-diameter sensory nerves and QoL, we used temperature thresholds in QST and the IENF density of a skin biopsy as biomarkers of small fiber sensory neuropathy.

In the QST, the abnormal rates for each functional examination compared with age-stratified norms were 54.7 % for the warm threshold of the foot (WT-foot), 33.4 % for the warm threshold of the hand (WT-hand), 15.1 % for the cold threshold of the foot (CT-foot), and 20.7 % for the cold threshold of the hand (CT-hand). Among these temperature thresholds, the warm threshold of the foot had the highest abnormal rate. For the IENF density analysis, the overall abnormal rate was 72.7 %, with a reduced IENF density in 80.0 % of the patients age <60 years and 66.7 % of the patients age >60 years.

**Fig. 1:**
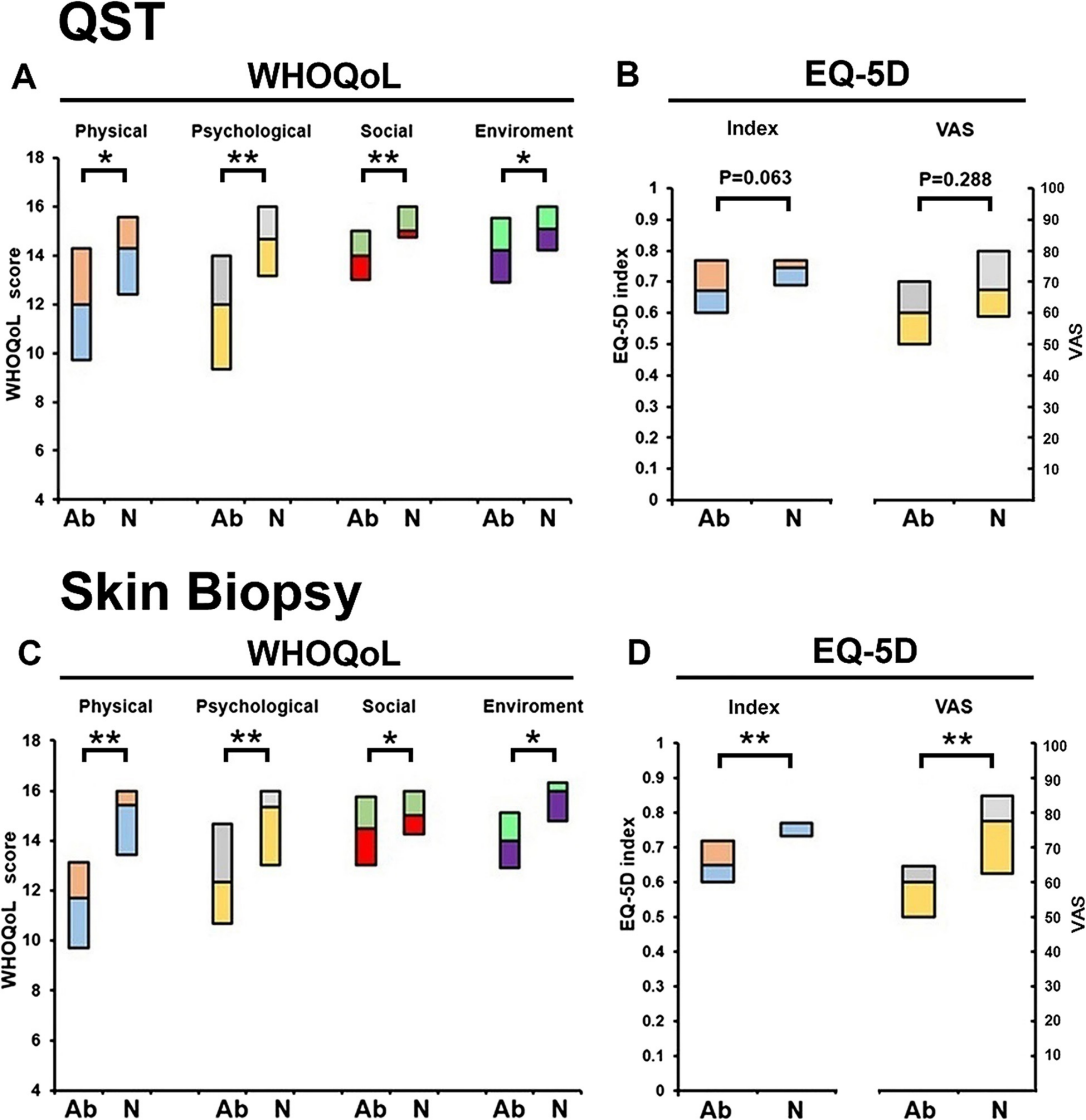
Small fiber impairment and quality of life (QoL) measurements. Box plot of small fiber sensory examination with QoL measures: quantitative sensory testing (QST in **a** and **b**) and skin biopsy (**c** and **d**) in WHOQoL (**a** and **c**) and EQ-5D (**b** and **d**). Patients were classified into two groups: normal (N) vs. abnormal (Ab) and each domain of QoL was analyzed accordingly. All domains of WHOQoL revealed significant association with QST results. Skin biopsy examination was related to all domains of WHOQoL and EQ-5D. (The bottom and top of the box are the first and third quartiles, * *P*<0.05,**P < 0.01,)

There were significant differences in all domains of the WHOQoL between patients with abnormal temperature thresholds and those with normal QST results (Fig. 1). The most significant differences, determined by statistical *p* values, were found in the physical domain (*p* = 0.014) and psychological domain (*p* = 0.007) (Additional file 1: Table S1).

The quantitative analysis of skin biopsies revealed that patients having a reduced IENF density, compared with patients having a normal IENF density, reported significantly lower scores in all of the WHOQoL domains (physical, psychological, social, and environment), in both of the EQ-5D categories (index and VAS), and higher scores in the 2 pain dimensions of the BPI-SF (‘pain during the previous 1 week’ and ‘interference dimension’) (Fig. 1 and Additional file 1: Table S1).

When the QST and skin biopsy results were combined as criteria for small fiber impairment (i.e., at least one abnormal temperature threshold or reduced IENF density), patients with small fiber impairment had significantly lower scores in (1) all domains of the WHOQoL, (2) the VAS category of the ED-5D, and (3) the interference dimension of the BPI-SF (Table 3).

**Table 3:** Comparison of patients’ quality of life (Mean ± SD) between normal and abnormal groups

	QST			IENFD		
	Normal	Abnormal	*P*	Normal	Abnormal	*P*
WHOQoL						
Physical domain	14.0 ± 2.0	11.9 ± 2.7	0.014	15.0 ± 2.0	11.5 ± 2.5	0.0005
Psychological domain	14.2 ± 2.2	11.9 ± 2.8	0.007	14.8 ± 2.3	12.1 ± 2.7	0.006
Social domain	15.6 ± 1.7	14.2 ± 1.8	0.006	15.1 ± 1.9	14.3 ± 2.4	0.048
Environment domain	15.2 ± 1.4	13.8 ± 2.2	0.030	15.5 ± 1.6	13.9 ± 2.3	0.011
EQ-5D						
Index	0.72 ± 0.09	0.68 ± 0.14	0.063	0.75 ± 0.04	0.68 ± 0.15	0.008
VAS	65 ± 20	62 ± 15	0.288	76 ± 13	57 ± 15	0.002
BPI-SF						
1 week	4.32 ± 1.67	5.03 ± 1.58	0.148	3.50 ± 1.57	4.78 ± 1.56	0.033
Now	3.91 ± 2.00	5.06 ± 1.88	0.100	3.75 ± 1.66	4.56 ± 2.26	0.284
Interference	2.44 ± 1.86	4.36 ± 2.02	0.005	1.89 ± 1.64	4.21 ± 2.28	0.007

WHOQoL domain scores range from 4 to 20. EQ-5D Index scores range from 0 to 1, and VAS scores range from 0 to 1000. BPI-SF scores ranged from 0 (not painful) to 10 (most painful)

In the facet-level analysis of WHOQoL, Pain and discomfort (F1), Mobility (F9) and Activities of daily living (F10) in physical domain of QoL was strongly associated with abnormal QST and IENF; these served as major contributing factors to QoL reduction (Additional file 1: Table S4).

Univariate linear regression analyses showed the physical domain of the WHOQoL correlated with 4 independent biomarkers of the QST (WT-hand, WT-foot, CT-hand, and CT-foot) (Additional file 1: Table S2). Multivariate linear regression was applied to investigate which QST parameters made major contribution to the 4 domains of the WHOQoL. The warm threshold at the foot was the only significant determinant for the all domains in the WHOQoL (Table 4).

**Table 4:** Multivariate linear regression analysis: relationship of temperature thresholds (warm threshold, WT, and cold threshold, CT) of quantitative sensory testing with each domain scores of WHOQoL

Domains	WT–hand			WT–foot			CT–hand			CT–foot			Age			Gender		
	Β	SE	*P*	β	SE	*P*	β	SE	*P*	β	SE	*P*	β	SE	*P*	β	SE	*P*
Physical	0.14	0.087336	0.117	0.359058	0.093182	0.000	–0.00	0.07	0.989	–0.04	0.060085	0.5176839	0.100208	0.024192	0.000	0.31	0.589971	0.602164
Psychological domain	0.11	0.111826	0.348527	0.351689	0.119311	0.005157	–0.08	0.083298	0.319	–0.04	0.076934	0.560088	0.090342	0.030976	0.005605	–0.08	0.755407	0.919013
Social	–0.02	0.081104	0.782848	0.279292	0.086533	0.002391	–0.04	0.060414	0.491	–0.02	0.055798	0.696015	0.067288	0.022466	0.004538	–0.07	0.547875	0.892668
Environment	0.07	0.088311	0.449768	0.202349	0.094222	0.037425	–0.04	0.065781	0.538	–0.04	0.060756	0.493875	0.059274	0.024462	0.020671	0.48	0.596555	0.426473

Abbreviation: *WT* warm threshold, *CT* cold threshold

We then examined the relation of the warm threshold of the foot and IENF density with the WHOQoL and pain characters using univariate linear regression analysis. The warm threshold at the foot demonstrated a linear correlation with the WHOQoL (physical, psychological and social domains) and BPI-SF (pain severity and pain interference dimensions). In contrast, only the IENF density demonstrated a linear association with the pain severity dimension (‘within 1 week’ and ‘right now’, β = -0.3, *p* = 0.003 and β = -0.31, *p* = 0.016, respectively) of the BPI-SF (Additional file 1: Table S3).

### Small fiber function: autonomic system

The abnormal rate of SSR and RRIV were 51.0% and 40.8.%, and in total 36.7 % (the number of abnormal SSR and RRIV) of the patients had autonomic dysfunction. In similar analyses for the RRIV and SSR examinations, only the abnormal RRIV demonstrated a significant association with a lower EQ5D index (*p* = 0.024) and a higher pain severity dimension (in the dimensions of within 1 week and right now) of the BPI-SF (*p* = 0.042 and *p* = 0.037, respectively) (Additional file 1: Table S1).

### Large fiber system

The abnormal rate of nerve conduction study was 59.6 %. For large fiber physiology, the parameters of the NCS, including motor nerves and sensory nerves, were analyzed according to their relationship with every aspects of QoL. Only patients with a lower SAP amplitude had a higher pain intensity in the BPI-SF than patients with a normal SAP amplitude (*p* = 0.038, Additional file 1: Table S1).

## Discussion

This study reports a significant reduction in the QoL with polyneuropathy according to global QoL assessment results, especially in the physical and psychological domains of the WHOQoL. Since weakness might be a confounding factor of QoL, we focused on the comparison of sensory parameters between large and small fiber impairment. Furthermore, the deterioration of QoL was correlated with biomarkers of small fiber neuropathy, i.e., thermal thresholds and IENF density. Presumably, pain resulting from neuropathy influences QoL, as shown in disease-specific instruments of the Norfolk QoL-DN [24–26]. A recent cohort study of elderly diabetic patients documented that some clinical parameters, including diabetes-related complications, smoking status, and Body-Mass-Index, predicted physical component scores of HRQoL [27]. There was, however, a lack of quantifiable and objective parameters that could demonstrate the relationships between impairment of small fiber thermonociceptive nerves and global QoL. To address this issue in our study, we used thermal thresholds and IENF density as biomarkers of small fiber impairment along with the autonomic function tests and NCS as biomarkers of autonomic and large fiber impairments, respectively. Only the temperature thresholds and IENF density were statistically significant in all domains of the WHOQoL among all parameters of polyneuropathy. Furthermore, the IENF density was also a significant determinant for the EQ-5D and BPI-SF. These 2 types of small fiber biomarkers had different modes of influences on QoL scores. Thermal threshold, especially the warm threshold on the dorsum foot, was linearly correlated with the WHOQoL domain scores. In contrast, IENF density appears to exert a non-linear association (i.e., threshold effect) on the WHOQoL and EQ-5D.

Previously QoL studies in polyneuropathy have mainly focused on diseases with large fiber impairment, such as Guillain-Barré syndrome (GBS), chronic inflammatory demyelinating polyneuropathy (CIDP), and Charcot-Marie-Tooth disease. Despite the documented reduction in QoL, no relationships among the electrophysiological measurements of the NCS, GBS disability score, and QoL score in the GBS were revealed [28]. In a randomized trial of CIDP, there was no significant correlation between the NCS parameters and QoL scores except for some significant variables after intravenous immunoglobulin treatment [29]. Rare reports of Charcot-Marie-Tooth disease indicated that there are some correlations between reduced NCS parameters (CMAP amplitude) and the physical and social functions of QoL [30].

The association between small fiber impairment and QoL have received less attention than the association between large fibers and QoL [25], even though small fiber neuropathy is prevalent in polyneuropathy of common etiologies (i.e diabetes mellitus, chronic kidney disease, drugs or toxin, and autoimmune) [1, 2]. Only one study specifically focused on small fiber neuropathy (based on reduced IENF density, abnormal thermal thresholds, and absence of large fiber impairment) was found and showed that there is reduced HRQoL in these patients [25]. These studies raise intriguing issues, such as (1) the influence of small fibers vs. large fibers on global QoL and (2) specific biomarkers of polyneuropathy as determinants of QoL. Given the complexity of polyneuropathy, small fiber impairment and large fiber neuropathy frequently coexist. It remains unclear, however, how small fiber neuropathy affects QoL. In this study, many aspects of QoL were highly correlated with thermal threshold or IENF density but were independent of the parameters of the NCS. These results indicate that the parameters of small fiber neuropathy are better predictors of QoL scores in polyneuropathy than the parameters of large fiber neuropathy. Thermal thresholds demonstrated linear correlations with the WHOQoL, and the warm threshold of the foot (WT-foot) was the most significant biomarker for the physical, psychological, and social domains of the WHOQoL. A previous study from our group reported a linear correlation between the thermal thresholds of the QST and the IENF density in a skin biopsy, and the foot warm threshold demonstrated the highest correlation with the IENF density in patients with type 2 diabetes [14]. The present study further demonstrates that the thermal thresholds (especially the warm threshold) at the foot and a skin biopsy not only reflect functional deficits and pathological changes in small fiber neuropathy, but they also predict a deterioration in QoL. These observations extend the clinical significance and applications of QST and a skin biopsy.

Despite the promising results of this study, several limitations are recognized. The comparison group for WHOQoL matched healthy subjects was retrieved from historical data. This study is a cross-sectional study performed at a single tertiary-care medical center and the etiologies of polyneuropathy in our studies patients were varied, which may limit the generalization of its findings to primary clinics. On the other hand, the diverse disease etiologies included in this study means that these observations might be applicable to polyneuropathy caused by different etiologies. Larger databases are necessary to identify the precise contributing factors leading to a reduced QoL, and may lead to the design of therapeutic approaches that improve treatment efficacy.

How will the QoL measures assessed in this study benefit patient care? Our validation of QoL promotes its use as a follow-up instrument in patients with small fiber neuropathy. Physicians can monitor disease progression by repeated measurements of HRQOL in their clinical practice. In addition, temporal changes of HRQoL along with the lifetime course of the illness can reflect patient’s functional state (i.e., physical, psychological and social domain) and utility [31], which are major outcomes of medical care. Using EQ-5D or WHOQoL-BREF instruments in clinical settings is relatively easy and very feasible; it takes about 10 minutes to complete the whole set of questionnaires based on our experiences. Our findings hint at the association between clinical measures of diagnosis tools (such as QST and the IENF density) at baseline and HRQoL measured in a cross-sectional survey. Further studies are needed to measure the longitudinal changes of HRQoL along withdifferent treatment modalities to investigate whether HRQoL is a predictor of long-term prognosis in patients, is a measure of disease burden in terms of savings of expected life years [32], or quality-adjusted life expectancy [33]. Expanding this study to estimate the lifetime healthcare expenditures [34] may facilitate the cost effectiveness analysis of multiple healthcare interventions.

## Conclusion

Our current study demonstrates biomarkers of small fiber impairment, including temperature thresholds in QST and the IENF density in a skin biopsy, were significant determinants of QoL in polyneuropathy patients. Furthermore, there was a linear correlation between the warm threshold of the foot and the QoL scores in the physical domain of the WHOQoL.

## Additional file

Additional file 1:
**Table S1**. Comparison of abnormal and normal patients’ quality of life (QoL). **Table S2**. Univariate linear regression analysis: relationship of temperature thresholds (warm threshold, WT, and cold threshold, CT) of quantitative sensory testing with quality of life (QoL). **Table S3**. Univariate linear regression analysis of IENF density and WT-foot with quality of life (QoL). **Table S4**. Facet-level comparison of WHOQoL. (DOC 184 kb)

## Abbreviations

WHOQoL: WHO quality of life-BREF
EQ-5D: European Quality of Life-5 Dimensions
VAS: Visual analogue scale for health-related QoL
BPI-SF: Brief pain inventory short form
QST: Quantitative sensory testing
WT: Warm threshold
CT: Cold threshold
IENFD: Intraepidermal nerve fiber density
SSR: Sympathetic skin response
RRIV: RR interval variation
