# Supplementary material for: Quality of life in polyneuropathy: association with biomarkers of small fiber impairment
Source: Health Qual Life Outcomes. 2015 Oct 15;13:169. doi: 10.1186/s12955-015-0363-9 (PMC4607005; doi:10.1186/s12955-015-0363-9)
Supplement: Additional file 1: — Table S1. Comparison of abnormal and normal patients’ quality of life (QoL). Table S2. Univariate linear regression analysis: relationship of temperature thresholds (warm threshold, WT, and cold threshold, CT) of quantitative sensory testing with quality of life (QoL). Table S3. Univariate linear regression analysis of IENF density and WT-foot with quality of life (QoL). Table S4. Facet-level comparison of WHOQoL. (DOC 184 kb) [file 12955_2015_363_MOESM1_ESM.doc]

**Additional file 1**

*World Health Organization Quality of Life instrument*

This Taiwanese version, adapted from the original instrument, has been thoroughly evaluated and has demonstrated internal consistency, test-and-retest reliability, discrimination, prediction, and validity [1]. The application methods, scoring procedures, and reference time point (during the last 2 weeks) followed the original procedures and protocols [2]. The gender- and age-matched healthy Taiwanese control subjects were randomly selected from the (1) National Health Interview Survey, a database of healthy individuals from a representative national sample [3] and the (2) Healthy Community-dwelling Elderly People, a survey of healthy elderly people who were over 65 years old and living in Taipei, Taiwan.

*European Quality of Life -5 Dimensions (EQ-5D)*

This study used the European Quality of Life -5 Dimensions (EQ-5D) as a measure of HRQoL and focuses on the choice and strength of preference for health outcomes by providing a single and complete indicator that represents the overall health status [4].

The 3-point scale of the EQ-5D could be transformed using the time trade off (TTO) method to a single index score, with a higher index score representing a better HRQoL. Studies on the EQ-5D have developed cross-cultural value sets that correspond to different countries, and the Taiwanese version of the EQ-5D has been validated [5].

Brief Pain Inventory *Short Form*

The Brief Pain Inventory short form (BPI-SF) was used to assess the severity and impact of pain on polyneuropathy patients. The BPI-SF provides information on the history, intensity, location, and quality of pain. Initially developed for cancer pain, the application of this validated questionnaire has been extended to non-cancer pain, including pain associated with diabetic neuropathy [6-8].

*Quantitative sensory testing*

The stimulator was applied to the skin for thermal stimuli. There was a good correlation between the 2 algorithms, method of limits and method of level. To eliminate concern for the reaction time on threshold determination, the values in the method of level were reported for analysis. The sensory analyzer delivered a stimulus set at a constant intensity and then delivered the next stimulus, which either increased or decreased at a fixed ratio depending on the response of the subject. The procedures were repeated until a predetermined difference in intensity was reached. The mean intensity of the final two stimuli was the threshold for the level method [9].

*Skin biopsy and quantitation of IENF density*

The IENF density as a pathologic parameter of skin innervation for small fiber neuropathy was quantified according to established protocols in a coded fashion, with examiners blind to the clinical information [10]. The results are expressed as the number of fibers per millimeter of epidermal length (fibers/mm). Normative values of IENF density for Taiwanese people have been reported and are similar to the values for other ethnic groups [11]. The 5th percentile values served as the cut-off values, 5.88 and 2.5 fibers/mm for people age ≤ 60 years and age > 60 years, respectively.

*Nerve conduction studies*

Nerve conduction studies (NCS) were performed with electromyography (Nicolet, Madison, WI, USA), following standardized methods. Amplitudes of the sensory action potential (SAP), compound muscle action potential (CMAP), nerve conduction velocity (NCV) and F-wave latency were recorded on motor (median, ulnar, peroneal and tibial) nerves and sensory (median, ulnar, sural and superficial peroneal) nerves. The value of each parameter was classified as normal vs. abnormal compared to the normative data with 2 standard deviations as the cut-off point. At least 2 nerves in the motor or sensory NCS with abnormal parameters were defined as abnormal in that category [12].

*Autonomic nervous system evaluation*

Autonomic tests include (1) sympathetic skin response (SSR) for sudomotor functions and (2) RR interval variation (RRIV) for cardiac vagal functions which evaluated exploiting beat-to-beat heart rate variation at rest and during deep breathing. The examinations were conducted according our established protocols and results were interpreted with age and gender- matched normative data of healthy controls [13]. Results of SSR in the sole were interpreted as present or absent, without quantitative evaluation due to variations in the latencies and amplitudes of SSR. A value of RRIV lower than mean-2SD of normative database was considered abnormal [14].

e-Reference

1. Huang IC, Wu AW, Frangakis C. Do the SF-36 and WHOQOL-BREF measure the same constructs? Evidence from the Taiwan population. Qual Life Res. 2006; **15**:15-24.
2. The WHOQOL Group. The World Health Organization Quality of Life Assessment (WHOQOL): development and general psycho-metric properties. Soc Sci Med 1998; **46**: 1569–1585.
3. Yao G, Wu CH. Factorial invariance of the WHOQOL-BREF among disease groups. Qual Life Res 2005; **14**: 1881-1888.
4. Rabin R, de Charro F. EQ-5D: a measure of health status from the EuroQol Group. Ann Med 2001; **33**: 337-343.
5. HY Lee, MC Hung, FC Hu, YY Chang, CL Hsieh, JD Wang. Estimating quality weights for EQ-5D (EuroQol-5 dimensions) health states with the time trade-off method in Taiwan. J Formos Med Assoc. 2013; **112**:699-706.
6. Ger LP, Ho ST, Sun WZ, et al. Validation of the Brief Pain Inventory in a Taiwanese population. J Pain Symptom Manage 1999; **18**:316–322.
7. Larue F, Colleau SM, Brasseur L, Cleeland CS. Multicentre study of cancer pain and its treatment in France. BMJ 1995; **310**: 1034–1037.
8. Tan G, Jensen MP, Thornby JI, Shanti BF. Validation of the Brief Pain Inventory for chronic nonmalignant pain. J Pain 2004; **5**: 133-137.
9. Pan CL, Tseng TJ, Lin YH, Chiang MC, Lin WM, Hsieh ST. Cutaneous innervation in Guillain-Barré syndrome: pathology and clinical correlations. Brain 2003; **126**: 386-397.
10. McCarthy BG, Hsieh ST, Stocks A, et al. Cutaneous innervation in sensory neuropathies: evaluation by skin biopsy. Neurology 1995; **45**: 1848-1855.
11. McArthur JC, Stocks A, Hauer P, Cornblath DR, Griffin JW. Epidermal nerve fiber density: normative reference range and diagnostic efficiency. Arch Neurol 1998; **55**: 1513-1520.
12. Nebuchennykh M, Løseth S, Lindal S, Mellgren SI. The value of skin biopsy with recording of intraepidermal nerve fiber density and quantitative sensory testing in the assessment of small fiber involvement in patients with different causes of polyneuropathy. J Neurol 2009; **256**: 1067-1075.
13. Lin YH, Hsieh SC, Chao CC, Chang YC, Hsieh ST. Influence of aging on thermal and vibratory thresholds of quantitative sensory testing. J Peripher Nerv Syst 2005; **10**: 269-281.
14. Ozgocmen S, Yoldas T, Yigiter R, Kaya A, Ardicoglu O, et al. R-R interval variation and sympathetic skin response in fibromyalgia. Arch Med Res 2006; **37**: 630-634.

**e-Table**

| **Additional file 1: Table S1. Comparison of abnormal and normal patients’ quality of life (QoL)** | | | | | | | | | | | |
| --- | --- | --- | --- | --- | --- | --- | --- | --- | --- | --- | --- |
|  | QST | | |  | IENFD | | |  | SSR | | |
| (Mean ± SD) | Normal | Abnormal | P |  | Normal | Abnormal | P |  | Normal | Abnormal | P |
| **WHOQoL** |  | | | | | | | | | | |
| Physical domain | 14.0 ± 2.0 | 11.9 ± 2.7 | 0.014 |  | 15.0 ± 2.0 | 11.5 ± 2.5 | 0.0005 |  | 12.7 ± 2.8 | 11.4 ± 2.4 | 0.091 |
| Psychological domain | 14.2 ± 2.2 | 11.9 ± 2.8 | 0.007 |  | 14.8 ± 2.3 | 12.1 ± 2.7 | 0.006 |  | 12.4 ± 3.2 | 12.3 ± 2.5 | 0.822 |
| Social domain | 15.6 ± 1.7 | 14.2 ± 1.8 | 0.006 |  | 15.1 ± 1.9 | 14.3 ± 2.4 | 0.048 |  | 14.5 ± 2.5 | 14.1 ± 1.8 | 0.452 |
| Environment domain | 15.2 ± 1.4 | 13.8 ± 2.2 | 0.030 |  | 15.5 ± 1.6 | 13.9 ± 2.3 | 0.011 |  | 14.1 ± 2.8 | 14.4 ± 1.7 | 0.882 |
| **EQ-5D** |  | | | | | | | | | | |
| Index | 0.72 ± 0.09 | 0.68 ± 0.14 | 0.063 |  | 0.75 ± 0.04 | 0.68 ± 0.15 | 0.008 |  | 0.68 ± 0.13 | 0.66 ± 0.10 | 0.191 |
| VAS | 65 ± 20 | 62 ± 15 | 0.288 |  | 76 ± 13 | 57 ± 15 | 0.002 |  | 63 ± 18 | 61 ± 14 | 0.772 |
| **BPI-SF** |  | | | | | | | | | | |
| 1 week | 4.32 ± 1.67 | 5.03 ± 1.58 | 0.148 |  | 3.50 ± 1.57 | 4.78 ± 1.56 | 0.033 |  | 4.33 ± 1.74 | 5.08 ± 1.58 | 0.237 |
| Now | 3.91 ± 2.00 | 5.06 ± 1.88 | 0.100 |  | 3.75 ± 1.66 | 4.56 ± 2.26 | 0.284 |  | 4.00 ± 2.11 | 5.12 ± 2.01 | 0.085 |
| Interference | 2.44 ± 1.86 | 4.36 ± 2.02 | 0.005 |  | 1.89 ± 1.64 | 4.21 ± 2.28 | 0.007 |  | 3.56 ± 2.34 | 4.58 ± 1.77 | 0.242 |

Abbreviation: WHOQoL = WHO quality of life-BREF, EQ-5D = European Quality of Life-5 Dimensions, BPI-SF = Brief Pain Inventory short form, QST = Quantitative sensory testing, IENFD = Intraepidermal nerve fiber density, SSR= sympathetic skin response, RRIV = RR interval variation.

**(continue)**

| RRIV | | |  | Motor system | | |  | Sensory system | | |
| --- | --- | --- | --- | --- | --- | --- | --- | --- | --- | --- |
| Normal | Abnormal | P |  | Normal | Abnormal | P |  | Normal | Abnormal | P |
|  |  |  |  |  |  |  |  |  |  |  |
| 12.8 ± 2.6 | 11.1 ± 2.6 | 0.053 |  | 13.0 ± 2.6 | 11.8 ± 3.2 | 0.121 |  | 12.8 ± 2.6 | 11.1 ± 2.6 | 0.063 |
| 12.5 ± 3.1 | 12.0 ± 2.6 | 0.453 |  | 12.4 ± 3.1 | 12.6 ± 2.5 | 0.995 |  | 12.5 ± 3.1 | 12.0 ± 2.6 | 0.830 |
| 14.7 ± 1.9 | 13.8 ± 2.4 | 0.218 |  | 14.4 ± 2.4 | 14.1 ± 2.1 | 0.723 |  | 14.7 ± 1.9 | 13.8 ± 2.4 | 0.631 |
| 14.4 ± 2.3 | 14.1 ± 2.3 | 0.815 |  | 14.5 ± 2.4 | 14.0 ± 2.1 | 0.213 |  | 14.4 ± 2.3 | 14.1 ± 2.3 | 0.327 |
|  |  |  |  |  |  |  |  |  |  |  |
| 0.70 ± 0.13 | 0.64 ± 0.08 | 0.024 |  | 0.71 ± 0.12 | 0.67 ± 0.12 | 0.252 |  | 0.71 ± 0.13 | 0.67 ± 0.12 | 0.112 |
| 64 ± 18 | 59 ± 12 | 0.218 |  | 65 ± 18 | 60 ± 15 | 0.420 |  | 65 ± 18 | 60 ± 16 | 0.508 |
|  |  |  |  |  |  |  |  |  |  |  |
| 4.21 ± 1.70 | 5.35 ± 1.42 | 0.042 |  | 4.36 ± 1.75 | 5.17 ± 1.39 | 0.091 |  | 4.13 ± 1.71 | 5.21 ± 1.43 | 0.038 |
| 4.00 ± 2.00 | 5.30 ± 2.05 | 0.037 |  | 4.18 ± 1.96 | 5.00 ± 2.07 | 0.121 |  | 3.91 ± 1.88 | 5.06 ± 2.07 | 0.062 |
| 3.66 ± 2.23 | 4.76 ± 2.30 | 0.143 |  | 3.50 ± 2.29 | 4.17 ± 2.26 | 0.245 |  | 3.05 ± 2.29 | 4.19 ± 1.99 | 0.052 |

| **Additional file 1: Table S2. Univariate linear regression analysis: relationship of temperature thresholds (warm threshold, WT, and cold threshold, CT) of quantitative sensory testing with quality of life (QoL)** | | | | | | | | | | | | | | | | | | | | | | | | | | | | |
| --- | --- | --- | --- | --- | --- | --- | --- | --- | --- | --- | --- | --- | --- | --- | --- | --- | --- | --- | --- | --- | --- | --- | --- | --- | --- | --- | --- | --- |
|  | WT-hand | | |  | WT-foot | | | | | |  | | CT-hand | | | | | |  | | CT-foot | | | | | | |  |
|  | β | SE | P |  |  | β | | SE | | P | |  | | β | | SE | | P | |  | | β | | SE | | P | |  |
| **WHOQoL** |  |  |  |  |  |  |  | |  | |  | |  | |  | |  | |  | |  | |  | |  | |  | |
| Physical | 0.23 | 0.07 | 0.002 |  |  | 0.33 | 0.08 | | <0.001 | |  | | 0.13 | | 0.05 | | 0.012 | |  | | 0.13 | | 0.05 | | 0.014 | |  | |
| Psychological domain | 0.10 | 0.08 | 0.223 |  |  | 0.24 | 0.09 | | 0.015 | |  | | 0.02 | | 0.06 | | 0.655 | |  | | 0.05 | | 0.06 | | 0.345 | |  | |
| Social | 0.01 | 0.06 | 0.839 |  |  | 0.16 | 0.07 | | 0.024 | |  | | 0.01 | | 0.04 | | 0.101 | |  | | 0.04 | | 0.04 | | 0.356 | |  | |
| Environment | 0.06 | 0.06 | 0.297 |  |  | 0.13 | 0.07 | | 0.083 | |  | | 0.01 | | 0.04 | | 0.760 | |  | | 0.02 | | 0.04 | | 0.634 | |  | |
| **EQ-5D** |  |  |  |  |  |  |  | |  | |  | |  | |  | |  | |  | |  | |  | |  | |  | |
| Index | 0.01 | 0.01 | 0.009 |  |  | 0.01 | 0.004 | | 0.156 | |  | | 0.01 | | 0.01 | | 0.171 | |  | | 0.01 | | 0.01 | | 0.485 | |  | |
| VAS | 0.93 | 0.48 | 0.056 |  |  | 1.03 | 0.57 | | 0.078 | |  | | 0.52 | | 0.32 | | 0.110 | |  | | 0.22 | | 0.34 | | 0.510 | |  | |
| **BPI-SF** |  |  |  |  |  |  |  | |  | |  | |  | |  | |  | |  | |  | |  | |  | |  | |
| Within 1 week | -0.07 | 0.05 | 0.125 |  |  | -0.12 | 0.05 | | 0.034 | |  | | -0.01 | | 0.03 | | 0.678 | |  | | -0.01 | | 0.03 | | 0.913 | |  | |
| Right now | -0.08 | 0.06 | 0.168 |  |  | -0.15 | 0.07 | | 0.035 | |  | | -0.01 | | 0.04 | | 0.884 | |  | | -0.01 | | 0.04 | | 0.880 | |  | |
| Interference | -0.17 | 0.06 | 0.006 |  |  | -0.29 | 0.07 | | <0.001 | |  | | -0.08 | | 0.04 | | 0.057 | |  | | -0.07 | | 0.04 | | 0.115 | |  | |
| Abbreviation: WT = warm threshold, CT = cold threshold, WHOQoL = WHO quality of life-BREF, EQ-5D = European Quality of Life-5 Dimensions, BPI-SF = Brief Pain Inventory short form. | | | | | | | | | | | | | | | | | | | | | | | | | | | | |

| **Additional file 1: Table S3.Univariate linear regression analysis of IENF density and WT-foot with quality of life (QoL)** | | | | | | | |
| --- | --- | --- | --- | --- | --- | --- | --- |
|  | IENF density | | |  | WT-foot | | |
|  | β | SE | P |  | β | SE | P |
| **WHOQoL** | | | | | | | |
| Physical domain | 0.23 | 0.17 | 0.192 |  | 0.33 | 0.08 | <0.001 |
| Psychological domain | 0.10 | 0.18 | 0.561 |  | 0.24 | 0.09 | 0.015 |
| Social domain | 0.04 | 0.14 | 0.798 |  | 0.16 | 0.07 | 0.024 |
| Environment domain | 0.06 | 0.14 | 0.672 |  | 0.13 | 0.07 | 0.083 |
| **EQ-5D** | | | | | | | |
| Index | 0.01 | 0.01 | 0.388 |  | 0.01 | 0.004 | 0.156 |
| VAS | 0.77 | 1.05 | 0.462 |  | 1.03 | 0.57 | 0.078 |
| **BPI-SF** | | | | | | | |
| Within 1 week | -0.30 | 0.09 | 0.003 |  | -0.12 | 0.05 | 0.034 |
| Right now | -0.31 | 0.12 | 0.016 |  | -0.15 | 0.07 | 0.035 |
| Interference | -0.17 | 0.14 | 0.231 |  | -0.29 | 0.07 | <0.001 |
| Abbreviation: WHOQoL = WHO Quality of Life-BREF, EQ-5D = European Quality of Life-5 Dimensions, BPI-SF = Brief Pain Inventory short form, IENFD = Intraepidermal nerve fiber density, WT= warm threshold | | | | | | | |

| **Additional file 1: Table S4. Facet-level comparison of WHOQoL** | | | | | | | | |
| --- | --- | --- | --- | --- | --- | --- | --- | --- |
| **WHOQoL** | QST | | |  | IENFD | | |  |
| (Mean ± SD) | Normal | Abnormal | P |  | Normal | Abnormal | P |  |
| **Physical domain** |  | | | | | | | |
| F1. Pain and discomfort | 3.6 ± 0.9 | 3.0 ± 0.9 | 0.026 |  | 3.7 ± 0.9 | 2.9 ± 1.0 | 0.038 |  |
| F2. Energy and fatigue | 3.4 ± 0.9 | 3.0 ± 0.9 | 0.175 |  | 3.5 ± 0.7 | 2.9 ± 0.9 | 0.034 |  |
| F3. Sleep and rest | 3.1 ± 1.1 | 2.7 ± 1.1 | 0.317 |  | 3.4 ± 0.7 | 2.6 ± 1.1 | 0.024 |  |
| F9. Mobility | 3.4 ± 1.0 | 2.8 ± 0.8 | 0.030 |  | 3.6 ± 0.7 | 2.7 ± 1.0 | 0.005 |  |
| F10. Activities of daily living | 3.7 ± 0.7 | 3.0 ± 0.9 | 0.020 |  | 3.9 ± 0.5 | 2.9 ± 0.8 | 0.002 |  |
| F11. Dependence on medication | 3.4 ± 1.1 | 3.3 ± 1.0 | 0.546 |  | 3.9 ± 1.0 | 3.0 ± 1.0 | 0.013 |  |
| F12. Work capacity | 3.9 ± 0.9 | 3.1 ± 1.0 | 0.004 |  | 3.8 ± 1.0 | 3.1 ± 1.1 | 0.030 |  |
| **Psychological domain** |  | | | | | | | |
| F4. Positive feelings | 2.9 ± 1.0 | 2.5 ± 1.0 | 0.188 |  | 3.2 ± 1.0 | 2.4 ± 0.9 | 0.021 |  |
| F5. Thinking/ learning/ memory | 3.9 ± 0.9 | 3.1 ± 1.0 | 0.004 |  | 3.7 ± 0.6 | 3.0 ± 1.0 | 0.045 |  |
| F6. Self-esteem | 4.2 ± 1.3 | 3.1 ± 0.8 | 0.001 |  | 4.0 ± 0.6 | 3.3 ± 1.4 | 0.012 |  |
| F7. Bodily image and appearance | 3.7 ± 0.7 | 3.1 ± 1.0 | 0.036 |  | 3.7 ± 0.6 | 3.1 ± 1.0 | 0.058 |  |
| F8. Negative feelings | 3.5 ± 0.8 | 3.1 ± 1.0 | 0.389 |  | 3.8 ± 0.6 | 3.2 ± 1.0 | 0.148 |  |
| F24. Spirituality/ religion/ beliefs | 3.7 ± 0.9 | 3.0 ± 1.0 | 0.028 |  | 3.6 ± 0.9 | 3.2 ± 1.1 | 0.278 |  |

Abbreviation: WHOQoL = WHO quality of life-BREF, QST = Quantitative sensory testing, IENFD = Intraepidermal

nerve fiber density.

Facet scores range from 1 to 5.
